# A network analysis bridging the gap between the big five personality traits and burnout among medical staff

**DOI:** 10.1186/s12912-024-01751-0

**Published:** 2024-02-04

**Authors:** Yifei Wang, Lin Wu, Chang Liu, Kuiliang Li, Mei Wang, Tingwei Feng, Qingyi Wang, Wu Chao, Lei Ren, Xufeng Liu

**Affiliations:** 1 Department of Military Medical Psychology, Air Force Medical University, 169 Street, 710032 Xi’an, China; 2 BrainPark, Turner Institute for Brain and Mental Health, School of Psychological Sciences, Monash University, 3168 Clayton, Australia; 3 Department of Psychology, Army Medical University, 400038 Chongqing, China; 4 Department of infectious diseases, Juxian Hospital of Traditional Chinese Medicine, Shandong Traditional Chinese Medicine University, 23 Street, 276500 Rizhao, China; 5 Department of Foreign Language Teaching and Research of Basic Ministry, Air Force Medical University, 169 Street, 710032 Xi’an, China; 6 School of Nursing, Air Force Medical University, 169 Street, 710032 Xi’an, China; 7 Military Psychology Section, Logistics University of PAP, 300309 Tianjin, China; 8 Military Mental Health Services & Research Center, 300309 Tianjin, China

**Keywords:** Big five personality traits, Burnout, Medical staff, Network analysis

## Abstract

**Background:**

Burnout is a common issue among medical professionals, and one of the well-studied predisposing factors is the Big Five personality traits. However, no studies have explored the relationships between these traits and burnout from a trait-to-component perspective. To understand the specific connections between each Big Five trait and burnout components, as well as the bridging effects of each trait on burnout, we employed network analysis.

**Methods:**

A cluster sampling method was used to select a total of 420 Chinese medical personnel. The 15-item Chinese Big Five Personality Inventory-15 (CBF-PI-15) assessed the Big Five personality traits, while the 15-item Maslach Burnout Inventory-General Survey (MBI-GS) assessed burnout components. Network analysis was used to estimate network structure of Big Five personality traits and burnout components and calculate the bridge expected influence.

**Results:**

The study revealed distinct and clear relationships between the Big Five personality traits and burnout components. For instance, Neuroticism was positively related to Doubt significance and Worthwhile, while Conscientiousness was negatively related to Accomplish all tasks. Among the Big Five traits, Neuroticism displayed the highest positive bridge expected influence, while Conscientiousness displayed the highest negative bridge expected influence.

**Conclusions:**

The network model provides a means to investigate the connections between the Big Five personality traits and burnout components among medical professionals. This study offers new avenues for thought and potential targets for burnout prevention and treatment in medical personnel, which can be further explored and tested in clinical settings.

**Supplementary Information:**

The online version contains supplementary material available at 10.1186/s12912-024-01751-0.

## Background

Burnout is a syndrome referred to as an “occupational phenomenon” despite lacking a single definition [1, 2]. Maslach and Jackson categorized burnout into three components: emotional exhaustion, depersonalization, and low feelings of personal accomplishment, which refers to a sense of competence and successful achievement in one’s work [3]. Numerous studies have demonstrated that burnout affects various healthcare professionals [4–6], negatively impacting their physical and mental well-being. Adverse effects may include stress-related syndromes or illnesses such as depression, anxiety, perceived memory impairment, diabetes, and metabolic syndrome [7]. Medical errors, decreased productivity, early retirement, and a compromised work-life balance are common consequences of these negative impacts [8–10]. Given its prevalence and potential detrimental effects, understanding the underlying causes of burnout is crucial.

Personality traits have been found to contribute to the development of burnout [11]. Numerous studies conducted in the past decade have emphasized the importance of psychological factors and identified specific personality traits that either facilitate or act as barriers to the development of burnout [12]. More recently, researchers have utilized the five-factor model of personality traits, commonly known as the “Big Five” to explore the relationship between personality and burnout [13]. The Five Factor Theory, which breaks down personality into five fundamental components, is a widely accepted framework for measuring traits. The “Big Five” personality traits consist of Neuroticism (degree of emotional instability), Extraversion (degree of sociability and liveliness), Agreeableness (degree of interpersonal tendencies to approach or reject others), Conscientiousness (degree of self-control and self-determination), and Openness (degree of intellectual curiosity and aesthetic sensibility) [14].

Most of the reviewed studies indicate that individuals with higher levels of Neuroticism and lower levels of Extraversion, Agreeableness, Conscientiousness, and Openness are more prone to burnout [15]. Neuroticism, in particular, may contribute to burnout due to difficulties in managing emotions and impulses. Neurotic individuals commonly experience insecurity, anxiety, anger, and depressive symptoms [16], which hinder their ability to perform job tasks satisfactorily and act as an amplifying “filter” for negative events [17], thereby increasing the risk of burnout [18]. On the other hand, Agreeableness, which enables warm interpersonal interactions, may have a protective effect against burnout, preventing individuals from experiencing depersonalization [19]. While several studies have investigated the relationship between the Big Five personality traits and burnout, the results have been inconsistent. Although most studies have found a strong negative association between Openness and burnout, other studies have reported opposite correlations between Openness and the three dimensions of burnout [20–22]. Furthermore, while certain studies have found a positive correlation between burnout and Extraversion, the reasons for this contradictory finding remain unexplained [21]. Given these discrepancies, further clarification of the links between the Big Five personality traits and burnout is necessary.

In previous studies, the correlation between the Big Five personality traits and burnout has often been examined by categorizing burnout as a unitary concept or its three dimensions [23–25]. However, this kind of approach may have an influence on overlooking the heterogeneity of burnout and mask the varying correlations between different components and personality traits, leading to inconsistent results. Previous research has demonstrated that burnout could be viewed as an interactive system comprising various components [26, 27]. Recent studies utilizing a network model have identified specific psychological characteristics, such as mental well-being [26] and depression [28], that are specifically associated with individual burnout components. Therefore, adopting a component-based approach may provide a fresh perspective and enhance understanding of the relationships between the Big Five personality traits and burnout.

From the perspective of network, a psychological construct can be seen as a network of components (nodes) and the interactions (edges) between them [29]. Depending on the strength of their direct connections (edge weights), nodes may either reinforce or inhibit each other in a network model incorporating the Big Five personality traits and burnout components. Network analysis offers a direct examination of the relationships between individual components and their predisposing factors, presenting an insightful visualization of these associations that traditional statistical models do not provide [30–32]. By examining the network structure, researchers can gain a clear understanding of which Big Five personality traits are closely linked to each of the burnout components. Additionally, network analysis offers new metrics for assessing the potential effects of significant predisposing factors on component communities [33]. For the community of burnout components, the bridge expected influence could specifically measure the extent to which each Big Five personality trait activates or deactivates the community, transmitting positive or negative effects [33]. This knowledge could be crucial in identifying prospective personality targets for burnout prevention and intervention.

The present study utilizes network analysis to compare the Big Five personality traits with burnout at the trait-to-component level. Our objectives are to investigate: (1) the specific connections between the Big Five personality traits and burnout components, and (2) the bridging effects of each Big Five personality trait on the cluster of burnout components by examining the bridge expected influence of each trait. We hypothesize that Neuroticism will activate the community of burnout components, while Conscientiousness will deactivate it.

## Methods

### Participants

Between April 16 and April 18, 2021, paper and pencil examinations were administered to collect data. The study participants consisted of 458 medical professionals from Xijing Hospital in the Chinese province of Shaanxi. Prior to participation, all participants provided informed consent. The investigation began with the collection of demographic information. 38 participants were excluded from the study for failing two honesty checks or providing incorrect answers to the demographic questions.

### Measures

#### Big five personality traits

The Chinese Big Five Personality Inventory-15 (CBF-PI-15) [34, 35] was used to assess the five dimensions of the Big Five personality traits. Each subscale (Neuroticism, Conscientiousness, Agreeableness, Openness, and Extraversion) included three items. Participants were asked to rate their responses on a six-point Likert-type scale, ranging from 1 (“disagree strongly”) to 6 (“agree strongly”). Sample items include “I often feel disturbed (Neuroticism)”, “One of my characteristics is doing things logically and orderly (Conscientiousness)”, “I think most people are well-intentioned (Agreeableness)”, “I’m a person who loves to take risks and break the rules (Openness)”, and “I like to go to social and recreational parties (Extraversion)”. With good reliability and validity (such as convergent, discriminant, and criterion-related validity) [34], the CBF-PI-15 has been widely adopted in previous studies (e.g., [36, 37]). The internal consistency values for each subscale were as follows: Neuroticism (0.83), Conscientiousness (0.75), Agreeableness (0.70), Openness (0.88), and Extraversion (0.70).

#### Burnout components

The Maslach Burnout Inventory-General Survey (MBI-GS) was first developed by American social psychologists Maslach and Jackson and was utilized to measure occupational burnout [38]. Each question is scored on a scale ranging from 0 (never) to 6 (very frequently), and the sum of these scores reflects the level of burnout. Li and colleagues found that one item in the cynicism dimension of the MBI-GS showed a significant cross load through exploratory factor analysis. Therefore, they recommended excluding this item for a better version of the MBI-GS (Chinese version) [39]. The Chinese version of the MBI-GS was usually adopted for its localization characteristics, as well as its strong reliability and validity. In the present study, the Chinese version of MBI-GS was adopted to investigate burnout among medical workers. This Chinese translation of the MBI-GS used in this study comprises 15 items divided into three dimensions: emotional exhaustion (items 1–5), depersonalization (items 6–9), and low feelings of personal accomplishment (items 10–15; reverse scoring). With good reliability and validity (such as construct validity) [39], the Chinese version of MBI-GS has been widely used in previous research (e.g., [40, 41]). The Cronbach’s alpha coefficient for the MBI-GS in this study was 0.93.

### Network analysis

The target trait-to-component network was estimated through graphical Least Absolute Shrinkage and Selection Operator based on Extended Bayesian Information Criterion criterium (hyperparameter gamma = 0.5) [42]. Within the network, edges depict the partial (Spearman) correlation between two nodes after controlling for all other nodes [42, 43]. The Fruchterman-Reingold algorithm [44] and R-package *qgraph* was used for the network construction and visualization [45].

Bridge expected influence was computed for each node in the target trait-to-component network by R-package *networktools* [33]. Higher positive/negative bridge expected influence value implies greater ability for activating/deactivating the other communities [33]. The communities were pre-defined: one community is Big Five personality traits (five nodes) and the other community is burnout components (fifteen nodes).

The network robustness test was conducted through R-package *bootnet* [29]. The accuracy of edge weights was examined by plotting the 95% confidence interval using 1,000 bootstrap samples and calculating bootstrapped difference tests for edge weights. The stability of bridge expected influence was assessed by computing the correlation stability (CS)-coefficient via a case-dropping bootstrap approach using 1,000 bootstrap samples and calculating bootstrapped difference tests for bridge expected influence. The ideal CS-coefficient is above 0.5 and should not be below 0.25 [29].

## Results

### Descriptive data analysis

The final sample comprised 221 nurses (female = 213) and 199 doctors (female = 130), all aged between 22 and 50 years (mean = 32.74, SD = 5.37). Table 1 presents the demographic characteristics of the participants. Table 2 displays the abbreviations, mean scores, standard deviations and bridge expected influence for each variable used in the current network analysis.

**Table 1:** Demographic characteristics of the participants

Characteristics	Variables	N / M	% / SD
Profession	Doctor	199	47.4
	Nurse	221	52.6
Gender	Female	343	81.7
	Male	77	18.3
Marriage	Married	304	72.4
	Single or divorced	116	27.6
Educational background	Undergraduate or less	269	64.0
	Postgraduate or more	151	36.0
Working years	<= 5	135	32.2
	6–10	150	35.6
	> 10	135	32.2
Job title	Junior	237	56.4
	Middle	163	38.8
	Senior	20	4.8
Age	18–30	155	36.9
	31–40	229	54.5
	41–50	34	8.6

Abbreviations: N: number; M: mean; SD: standard deviation

**Table 2:** Abbreviations, mean scores, standard deviations and bridge expected influence for each variable selected in the current network analysis

Variables	Abbr	M	SD	BEI^a^
Traits of Big Five Personality				
Agreeableness	Agr	15.20	2.26	-0.15
Conscientiousness	Con	14.47	2.46	-0.27
Extraversion	Ext	11.73	2.99	-0.24
Neuroticism	Neu	7.96	3.40	0.51
Openness	Ope	10.94	3.30	-0.06
Components of Burnout				
I feel emotionally drained from my work (Emotionally drained)	B1	1.59	1.35	-0.05
I feel used up at the end of the day (Used up)	B2	1.80	1.52	0
I feel tired when I get up in the morning and have to face another day at work (Tired)	B3	1.18	1.37	-0.04
Working with people all day is a real strain for me (Strain)	B4	1.23	1.39	0.02
I feel burned out from my work (Burned out)	B5	0.75	1.15	0.002
I have become more callous toward work since I took this job (Callous)	B6	0.70	1.10	-0.07
I have become less enthusiastic about my work (Less enthusiastic)	B7	0.81	1.15	-0.01
I doubt the significance of my work (Doubt significance)	B8	0.65	1.03	0.15
I have become more and more indifferent in the contribution of my job (Indifferent)	B9	0.60	1.06	-0.004
I deal effectively with the problems of clients^*^ (Effectively)	B10	1.07	1.22	-0.006
I feel that I am contributing to my company^*^ (Contributing)	B11	1.15	1.30	0
In my opinion, I am good at my job^*^ (Good at job)	B12	1.05	1.21	0.05
I feel very happy when I accomplish some tasks of my job^*^ (Happy)	B13	0.93	1.22	-0.17
I have accomplished many worthwhile things in this job^*^ (Worthwhile)	B14	1.28	1.34	0.03
I am confident that I can accomplish all tasks effectively^*^ (Accomplish all tasks)	B15	1.05	1.25	-0.13

Abbreviations: Abbr: Abbreviation; M: mean; SD: standard deviation; BEI: bridge expected influence

^*^ Reverse scoring item; ^a^ Raw score

### Network structure

**Fig. 1:**
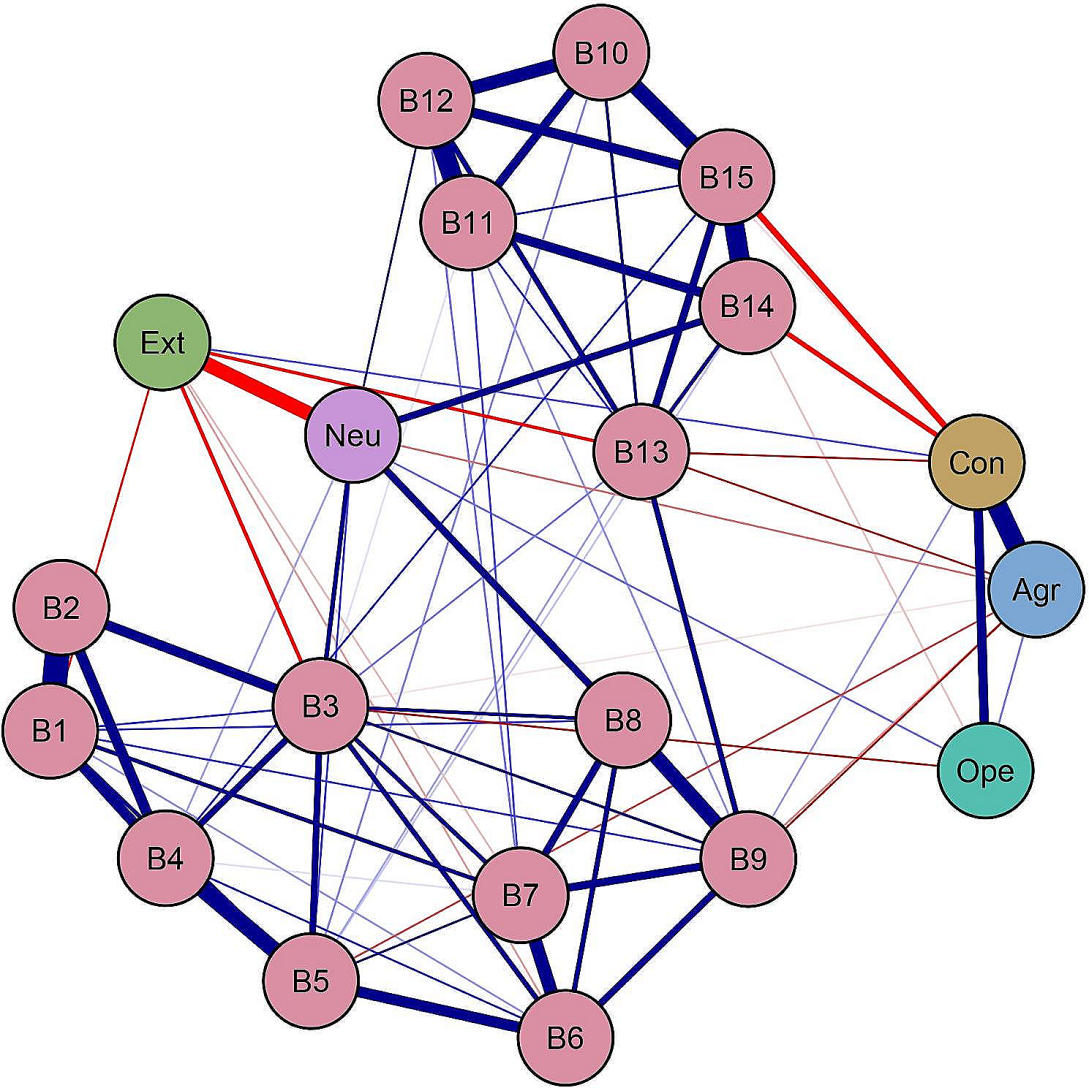
Network structure of Big Five personality traits and burnout. Blue edges represent positive connections, red edges represent negative connections. A total description of nodes of Big Five personality traits and burnout components could be seen in Table 2

Figure 1 represents the estimated network, which retained a total of 23 between-community edges (30.67%) with non-zero edge weights (ranging from − 0.13 to 0.15) out of 75 potential between-community edges. Table S1 (in supplementary materials) shows all edges weights within the final network. Among the fifteen burnout components, six exhibited positive correlations with Neuroticism (weights ranging from 0.02 to 0.15). The strongest edge existed between Neuroticism and Doubt significance (B8; edge weight = 0.152). The second-strongest edge connected Neuroticism and Worthwhile (B14; edge weight = 0.150). Conscientiousness demonstrated connections with five burnout components (out of 15), with four negative edges and one positive edge (weights ranged from − 0.13 to 0.02). The burnout component Accomplish all tasks (B15; edge weight = -0.13) exhibited the strongest negative relationship with Conscientiousness. The second strongest negative edge connected Conscientiousness and Worthwhile (B14; edge weight = -0.11). Among the fifteen burnout components, five showed negative correlations with Agreeableness (weights ranging from − 0.05 to -0.004). Two burnout components (out of 15) demonstrated negative links with Openness, with edge weights of -0.05 and − 0.01, respectively. Similarly, five burnout components (out of 15) were negatively associated with Extraversion, with weights ranging from − 0.08 to -0.01. The edge between Extraversion and Tired exhibited the strongest negative correlation (B3; edge weight = -0.084). The second-strongest negative edge connected Extraversion and Happy (B13; edge weight = -0.076). Figure S1 (Supplementary Material) showed the bootstrapped 95% confidence intervals for edge weights. The bootstrapped difference test for edge weights was displayed in Figure S2 (Supplementary Material).

**Fig. 2:**
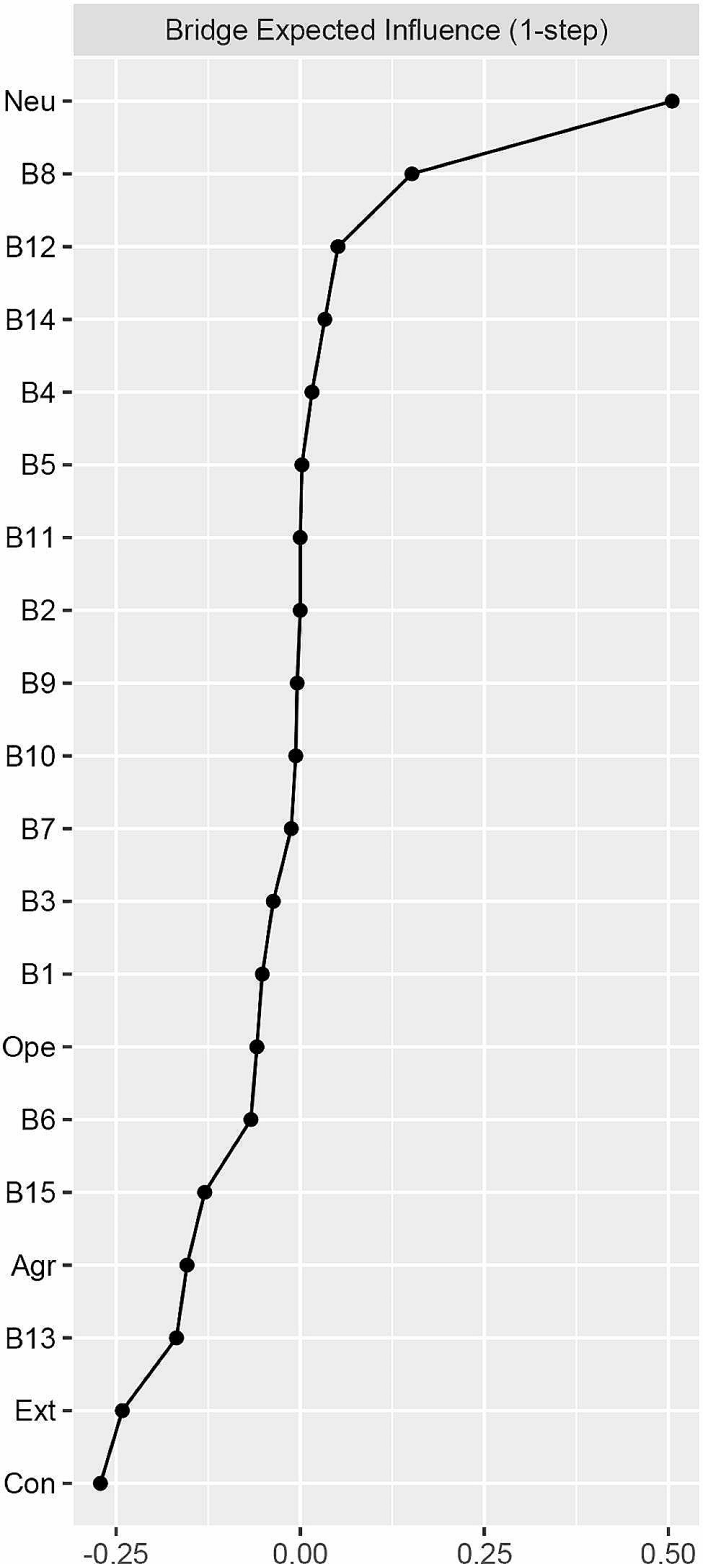
Bridge expected influence plot. A total description of nodes of Big Five personality traits and burnout components could be seen in Table 2

Table 2; Fig. 2 illustrate the raw bridge expected influence values. Neuroticism exhibited the highest positive bridge expected influence among all nodes (value = 0.51), while Conscientiousness showed the highest negative bridge expected influence (value = -0.27). Figure S3 (in Supplementary Material) demonstrated the adequate stability of the bridge expected influence, with a CS-coefficient value of 0.75 exceeding 0.50. The bootstrapped difference test (Figure S4 in the Supplementary Material) revealed differences in the bridge expected influence among nodes.

## Discussion

The present study is the first to utilize a trait-to-component network approach to explore the connections between the Big Five personality traits and burnout components. In line with our initial objective, we uncovered several distinct between-community connections, both positive and negative, such as Neuroticism-Doubt significance (B8), Conscientiousness-Accomplish all tasks (B15), and Extraversion-Tired (B3). The strongest positive association was observed between Neuroticism and Doubt significance (B8). Our second objective and study hypotheses were supported by the results of bridge expected influence, which revealed that Conscientiousness and Extraversion deactivate the burnout components community while Neuroticism activates it. We also demonstrated that Agreeableness and Openness may deactivate the burnout components community.

The strongest positive edges between communities were observed between Neuroticism and Doubt significance (B8), while the second-strongest edges were found between Neuroticism and Worthwhile (B14). These two edges illustrate the association between Neuroticism and depersonalization, as well as low emotions of personal accomplishment. Neuroticism, characterized by worry, insecurity, depression, fear, and apprehension [16, 46, 47], often leads individuals to employ avoidance and diversion as coping mechanisms [48]. In demanding and highly competitive careers, such behavior is likely to result in higher levels of depersonalization and reduced personal accomplishment [49, 50]. This finding is consistent with previous research on burnout among medical professionals [50–52]. Most connections between Conscientiousness and burnout, such as those between Conscientiousness and Accomplish all tasks (B15) and Worthwhile (B14), were found to be negative. Conscientious individuals, known for their ability to manage and organize their work and time, are adept at employing efficient coping mechanisms that keep their focus on problem-solving in stressful situations [14]. This argument aligns with earlier research suggesting that Conscientiousness facilitates individuals’ perception of professional efficacy [53]. We also discovered an intriguing positive link between Conscientiousness and Indifferent (B9). This relationship may be attributed to the occupational peculiarities of medical staff; under increased pressure resulting from deteriorating doctor-patient relationships, medical staff may view it as their duty to provide for patients while avoiding excessive emotional involvement. Nevertheless, further investigations are needed to provide a more detailed explanation for this finding. Furthermore, the final network structure revealed that the majority of components in the burnout community were positively connected. Three edges with the highest weights within the burnout community were Emotionally drained-Used up (B1-B2), Contributing-Good at job (B11-B12), and Worthwhile-Accomplish all tasks (B14-B15). These results about strongest edges were similar to our previous studies on the network structure of burnout among medical staff and Chinese nurses [26, 27].

The bridge centrality of nodes may shed light on the specific roles played by each of the Big Five personality traits in the context of burnout [54, 55]. Nodes with higher bridge expected influence values are more likely to activate the burnout components community. Thus, from the perspective of the Big Five personality traits, this provides empirical evidence for early detection and intervention of medical staff burnout. Specifically, Neuroticism exhibits a high positive bridge expected influence value, suggesting that it effectively activates the burnout component community. This finding is consistent with a previous study that utilized network analysis to examine the bridging effects of each Big Five personality trait on the symptom community of problematic smartphone use and found that Neuroticism had the highest positive bridge centrality [56]. Individuals with high levels of Neuroticism often experience heightened levels of stress, tend to magnify the seriousness of threats, and underestimate their own capabilities. On the other hand, Conscientiousness exhibits the highest negative value of bridge expected influence, indicating its potential to effectively deactivate the burnout components community. Bridge nodes have been identified as crucial intervention targets since addressing them could modify the co-occurring phenomenon of communities [33]. Therefore, addressing medical staff burnout may involve reducing Neuroticism and enhancing Conscientiousness.

### Limitations

Although the present study employs a novel component-based approach, namely network analysis, to explore the connections between the Big Five personality traits and burnout components among medical staff, there are several limitations that warrant consideration. Firstly, the theoretical foundation of this study assumes that personality characteristics can impact burnout, and the findings were interpreted in light of the potential predictive pathways between personality traits and burnout. However, due to the cross-sectional design employed in this study, we cannot completely exclude the possibility that the Big Five personality traits may have changed as a consequence of experiencing burnout symptoms. Secondly, if alternative measurement scales are utilized for assessing the components, it is uncertain whether the network structure established in this study, based on the questionnaires employed, can be replicated. Moreover, since the instrument employed relies on self-reporting, response bias is inevitable, although future research could incorporate additional objective measurement techniques. Finally, the current study’s sample size selection was informed by the work of Epskamp et al. (2018), which suggest a minimum sample size of 210 for a 20-node network analysis [29]. Additionally, we calculated the CS-coefficient, adhering to recommended best practices for ensuring network stability. It’s important to note that while the CS-coefficient is optimal for this study, its determination was post hoc rather than a priori. Since our data collection, newer methodologies for a priori sample size estimation have emerged. Specifically, the method proposed by Constantin and colleagues indicates that a sample size of 3582 could achieve a sensitivity of 0.6 in 80% of cases [57]. This larger sample size is recommended for future studies aiming to replicate our findings.

## Conclusions

Future research should prioritize elucidating the potential significance of personality traits, encouraging early detection of at-risk people, and developing successful therapies [58]. The onset and progression of burnout are significantly influenced by personality factors [59]. Our investigation of the Big Five personality traits and burnout components among medical staff from a trait-to-component viewpoint is the first, as far as we are aware. Despite the aforementioned restrictions, this research has significant theoretical and clinical implications. On the one hand, we investigate the relationships between the Big Five personality traits and burnout’s components, which may enhance the relationship between the Big Five personality traits and burnout’s potential theoretical mechanism and offer a fine-grained understanding of how medical staff with various personalities influence various burnout’s components (potential pathways). However, when it comes to addressing the needs of lowering burnout in medical personnel, the findings on Neuroticism (with the highest positive bridge expected influence) and Conscientiousness (with the highest negative bridge expected influence) may have significant consequences.

### Electronic supplementary material

Below is the link to the electronic supplementary material.

Supplementary Material 1

Supplementary Material 2

## Data Availability

The datasets used and/or analyzed during the current study are available from the corresponding author on reasonable request.
