# Supplementary material for: A network analysis bridging the gap between the big five personality traits and burnout among medical staff
Source: BMC Nurs. 2024 Feb 4;23:92. doi: 10.1186/s12912-024-01751-0 (PMC10838458; doi:10.1186/s12912-024-01751-0)
Supplement: Supplementary file 1 — Supplementary Material 1 [file 12912_2024_1751_MOESM1_ESM.pdf]

Supplementary Materials

1. Table S1. All edges weights within the final network
2. Figure S1. Accuracy of edge weights
3. Figure S2. Bootstrapped difference test for edge weights
4. Figure S3. Stability of the bridge expected influence
5. Figure S4. Bootstrapped difference test for the bridge expected influence

Table S1. All edges weights within the final network

	Neu	Con	Agr	Ope	Ext	B1	B2	B3	B4	B5	B6	B7	B8	B9	B10	B11	B12	B13	B14	B15
**Neu**	0.00	0.00	-0.03	0.00	-0.29	0.00	0.00	0.10	0.02	0.04	0.00	0.00	0.15	0.00	0.00	0.00	0.05	0.00	0.15	0.00
**Con**	0.00	0.00	0.40	0.19	0.03	0.00	0.00	0.00	0.00	0.00	0.00	0.00	0.00	0.02	-0.01	0.00	0.00	-0.05	-0.11	-0.13
**Agr**	-0.03	0.40	0.00	0.02	0.00	0.00	0.00	0.00	0.00	-0.03	-0.05	0.00	0.00	-0.02	0.00	0.00	0.00	-0.04	0.00	0.00
**Ope**	0.00	0.19	0.02	0.00	0.03	0.00	0.00	-0.05	0.00	0.00	0.00	0.00	0.00	0.00	0.00	0.00	0.00	0.00	-0.01	0.00
**Ext**	-0.29	0.03	0.00	0.03	0.00	-0.05	0.00	-0.08	0.00	0.00	-0.02	-0.01	0.00	0.00	0.00	0.00	0.00	-0.08	0.00	0.00
**B1**	0.00	0.00	0.00	0.00	-0.05	0.00	0.48	0.04	0.21	0.09	0.02	0.09	0.04	0.04	0.00	0.00	0.00	0.00	0.00	0.00
**B2**	0.00	0.00	0.00	0.00	0.00	0.48	0.00	0.21	0.22	0.00	0.00	0.00	0.00	0.00	0.00	0.00	0.00	0.00	0.00	0.00
**B3**	0.10	0.00	-0.004	-0.05	-0.08	0.04	0.21	0.00	0.14	0.14	0.11	0.10	0.08	0.06	0.00	0.01	0.00	0.02	0.00	0.00
**B4**	0.02	0.00	0.00	0.00	0.00	0.21	0.22	0.14	0.00	0.32	0.05	0.01	0.00	0.00	0.00	0.00	0.00	0.00	0.00	0.05
**B5**	0.04	0.00	-0.03	0.00	0.00	0.09	0.00	0.14	0.32	0.00	0.22	0.06	0.00	0.00	0.02	0.00	0.00	0.02	0.01	0.00
**B6**	0.00	0.00	-0.05	0.00	-0.02	0.02	0.00	0.11	0.05	0.22	0.00	0.27	0.11	0.15	0.00	0.00	0.00	0.00	0.00	0.00
**B7**	0.00	0.00	0.00	0.00	-0.01	0.09	0.00	0.10	0.01	0.06	0.27	0.00	0.16	0.17	0.00	0.03	0.02	0.00	0.00	0.00
**B8**	0.15	0.00	0.00	0.00	0.00	0.04	0.00	0.08	0.00	0.00	0.11	0.16	0.00	0.29	0.00	0.00	0.00	0.00	0.00	0.00
**B9**	0.00	0.02	-0.02	0.00	0.00	0.04	0.00	0.06	0.00	0.00	0.15	0.17	0.29	0.00	0.00	0.02	0.00	0.12	0.00	0.00
**B10**	0.00	-0.01	0.00	0.00	0.00	0.00	0.00	0.00	0.00	0.02	0.00	0.00	0.00	0.00	0.00	0.18	0.27	0.07	0.00	0.31
**B11**	0.00	0.00	0.00	0.00	0.00	0.00	0.00	0.01	0.00	0.00	0.00	0.03	0.00	0.02	0.18	0.00	0.39	0.04	0.20	0.05
**B12**	0.05	0.00	0.00	0.00	0.00	0.00	0.00	0.00	0.00	0.00	0.00	0.02	0.00	0.00	0.27	0.39	0.00	0.12	0.00	0.21
**B13**	0.00	-0.05	-0.04	0.00	-0.08	0.00	0.00	0.02	0.00	0.02	0.00	0.00	0.00	0.12	0.07	0.04	0.12	0.00	0.09	0.16
**B14**	0.15	-0.11	0.00	-0.01	0.00	0.00	0.00	0.00	0.00	0.01	0.00	0.00	0.00	0.00	0.00	0.20	0.00	0.09	0.00	0.39
**B15**	0.00	-0.13	0.00	0.00	0.00	0.00	0.00	0.00	0.05	0.00	0.00	0.00	0.00	0.00	0.31	0.05	0.21	0.16	0.39	0.00

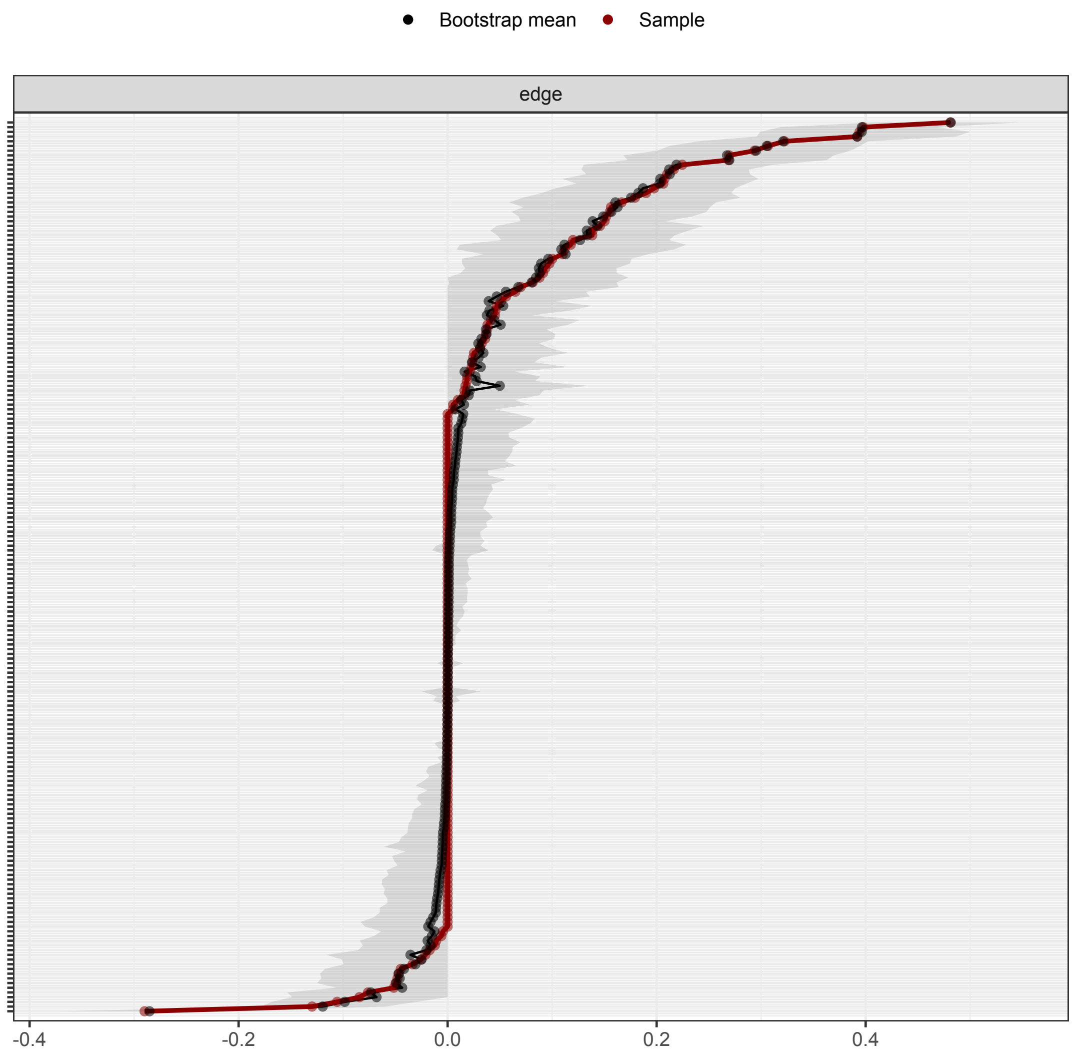

Figure S1. Accuracy of edge weights

Note: The red line depicts the sample edge weights and the gray bar depicts the bootstrapped confidence interval.

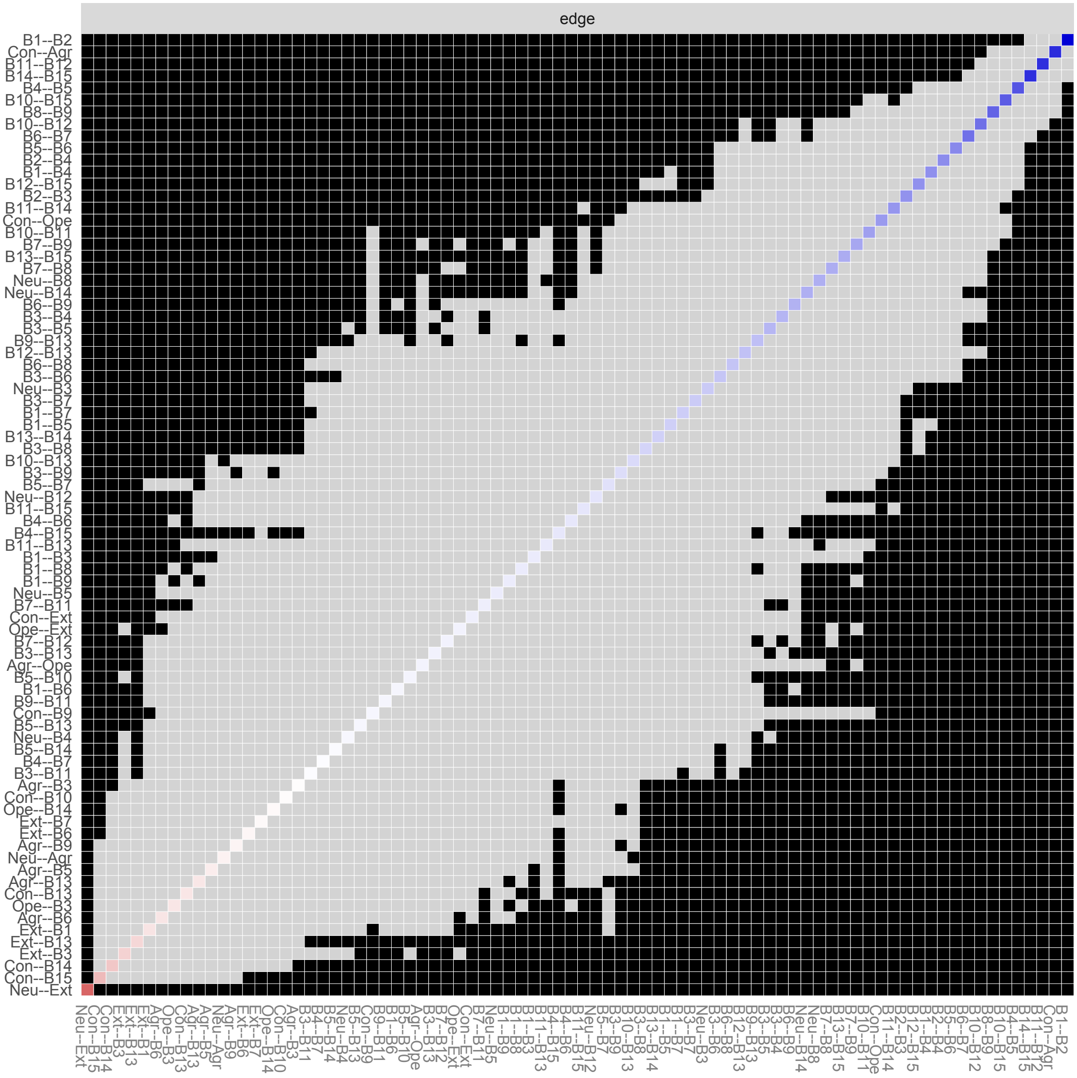

Figure S2. Bootstrapped difference test for edge weights

Note: Gray boxes indicate edge weights that do not differ significantly from one another, while black boxes indicate edge weights that do differ significantly. Blue and red boxes on the diagonal correspond to edge weights with positive and negative correlations, respectively.

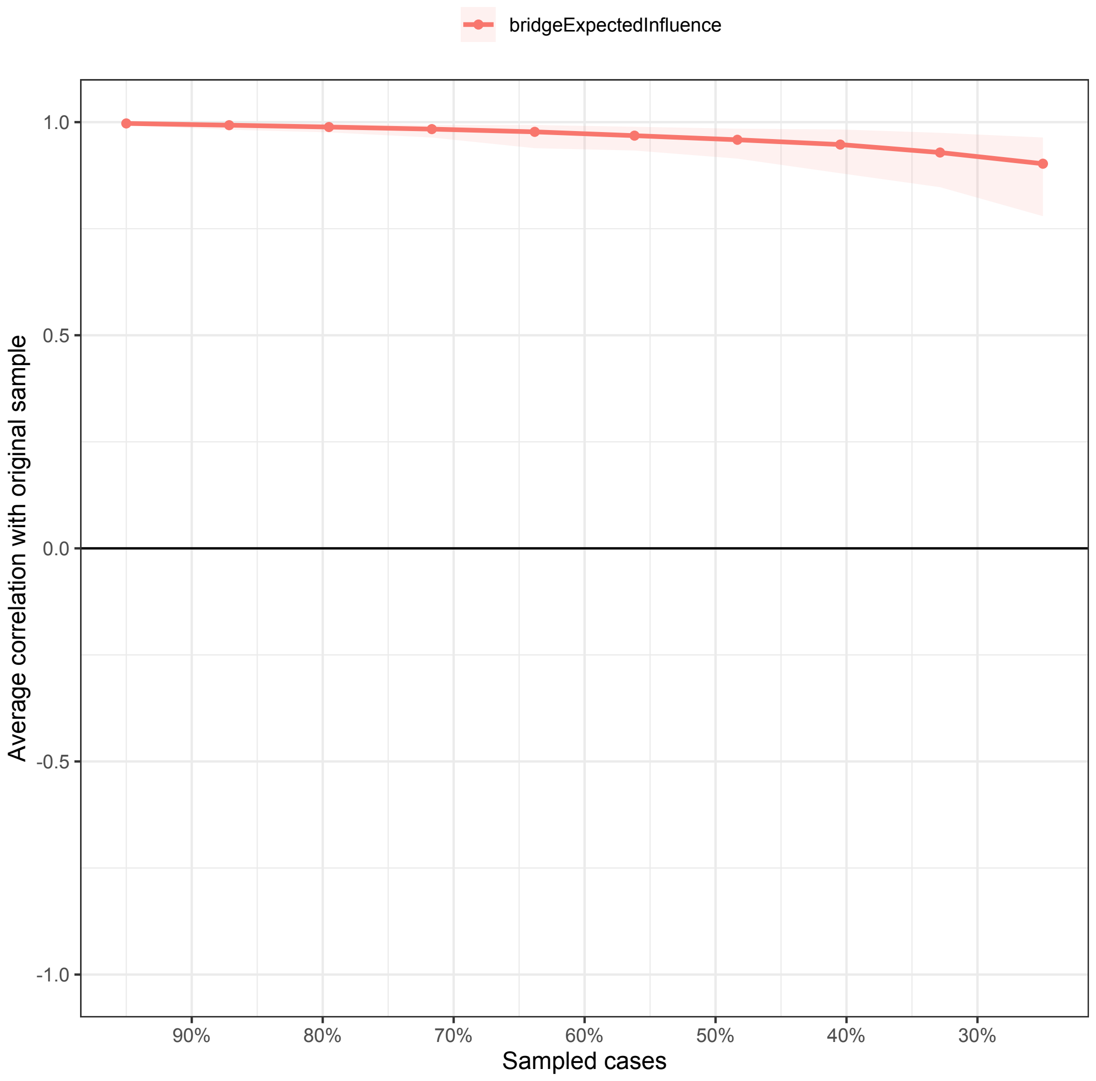

Figure S3. Stability of the bridge expected influence values

Note: The red bar represents the average correlation between bridge expected influence.

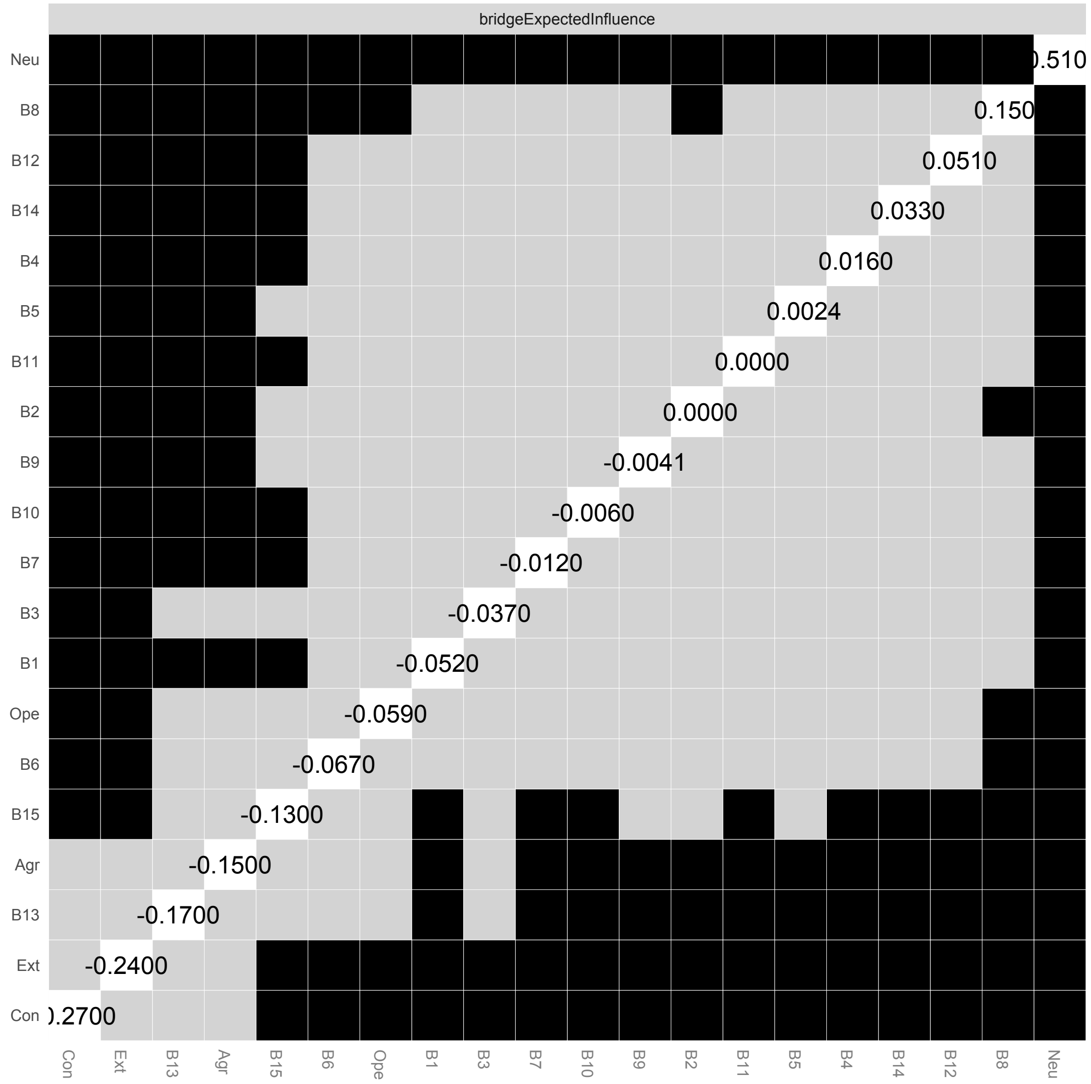

Figure S4. Bootstrapped difference test for the bridge expected influence values

Note: Gray boxes indicate the bridge expected influence that do not differ significantly from one another, while black boxes indicate the bridge expected influence that do differ significantly. The number in the white boxes (i.e., diagonal line) represent the value of the bridge expected influence.
